# Study of F-wave Components in a Control Population of Young Adults

**DOI:** 10.7759/cureus.81059

**Published:** 2025-03-23

**Authors:** Sangeeta Gupta, Ramji Singh

**Affiliations:** 1 Physiology, All India Institute of Medical Sciences, Gorakhpur, IND; 2 Physiology, All India Institute of Medical Sciences, Kalyani, IND

**Keywords:** chronodispersion, f/m amplitude ratio, f-response, f-wave, f-wave mean latency, f-wave minimum latency, height, latency, persistence

## Abstract

Introduction

The F-wave represents a delayed muscular response that occurs as a result of the antidromic activation of one or a few motoneurons ensuing the electrical stimulation of a peripheral nerve. F-wave response has various parameters, yet the primary application of F-waves is presently confined to latency measurement, with a particular emphasis on F-wave minimum latency. Our goal is to report F-wave data in a population of young healthy individuals, encompassing rarely examined components including F-wave/M-wave amplitude (F/M ratio), duration, chronodispersion, and persistence in both upper and lower extremity nerves.

Methods

F-response components were recorded from median, ulnar, tibial, and peroneal nerves in 100 healthy volunteers in the age group of 18-40 years. Linear regression analysis was conducted for the correlation of F-wave parameters with height and age. Gender comparison (unpaired t-test) and side-to-side comparison (paired t-test) were performed. A p-value of <0.05 was considered statistically significant.

Results

R2 values for the relationship of F-wave minimum latency with height for median, ulnar, tibial, and peroneal nerves were 0.755, 0.761, 0.739, and 0.650, respectively, suggesting a strong direct relation. F-wave minimum latency (mean value) increased by about 0.27±0.7 ms on the right side (as compared to the left) with statistical significance (p<0.001) (paired t-test) for the F-wave median study. Male participants had increased F-wave minimum latency: p<0.0001 and p<0.01 (unpaired t-test) for median (right and left sides, respectively) while p<0.05 for ulnar, tibial, and peroneal nerves (both sides). No other F-wave components exhibited significant variation in height, gender, and age.

Conclusion

The current research has established normal values for the various F-response components, including F-wave latencies, F-wave duration, F/M amplitude ratio, chronodispersion, and persistence in the young adult age group. Side difference reference values have also been established. A strong influence of height should be borne in mind during F-wave interpretation.

Reference values for the relatively scarcely studied F-wave component including F/M amplitude ratio, F-wave duration, F-wave persistence, and chronodispersion can be helpful in diversifying the clinical applicability of the F-response study.

## Introduction

The F-wave is a delayed muscular response caused by the antidromic activation of one or a few motoneurons subsequent to the electrical stimulation of a peripheral nerve [1,2]. Since its initial description and preliminary clinical research, the F-wave has been widely used to evaluate peripheral nerve lesions [3-5]. In recent years, the value of F-wave in generalized as well as localized peripheral nerve lesions has been well-established [6,7]. The F-wave uses supramaximal stimulation of a motor nerve and records compound muscle action potentials from a muscle supplied by that nerve, along the most proximal segment. F-waves are recorded as a compound F-wave population (CFP) under identical stimulus and recording conditions and follow the direct motor potential or the M-response. The consecutively recorded F-waves vary in latency, amplitude, duration, area, and morphology depending upon the individual's antidromically activated motor neurons.

F-wave parameters include the latency, F-wave/M-wave amplitude (F/M ratio), duration, persistence, and conduction velocity. Latency parameters for an F-wave record are three: the minimal, mean, and maximal latency. The minimal F-wave has the fastest conducting fibers, while the maximum has the slowest conducting fibers reflecting the longest latency. However, recording the mean latency value for a sequence of F-waves (FLmean) is statistically preferable and has the practical advantage of averting dependence on a single latency measurement [8]. Persistence (calculated by dividing the number of F-responses by the number of stimuli) shows variation depending on the muscle, i.e., highest for flexors of the arm and extensors of the legs [9]. Chronodispersion is the difference between the maximal and minimal latencies and indicates the scatter or dispersion of the relative latencies of statistically significant numbers of consecutively recorded F-waves [2].

F-waves provide valuable information about a wide range of motor disorders such as polyneuropathies and demyelinating conditions [10,11]. The study of F-waves is particularly useful for the diagnosis of proximal nerve lesions that would be otherwise inaccessible to other routine nerve conduction studies [12]. F-wave latency has proved to be a more sensitive indicator of a conduction abnormality than the maximal motor and sensory conduction velocities [4]. The F-wave serves as a sensitive measure for axonal polyneuropathy and radiculopathy and is used in the diagnosis of diabetic polyneuropathy (DPN), Guillain-Barré syndrome (GBS), and amyotrophic lateral sclerosis (ALS). The F-wave study has also been found to be sensitive in the early diagnosis of subclinical neuropathy in diabetes mellitus [6,13].

Various F-wave parameters have been found to be useful in the diagnostic evaluation of peripheral nerve disorder [14]. F-wave latency was found most sensitive in patients with DPN [15]. Median and tibial F-wave latencies have been suggested to provide the most reproducible measures for nerve conduction study, serving as one of the best measures for DPN [16]. Chronodispersion has been reported to be a sensitive marker of abnormalities in lumbosacral radiculopathies [17]. It is highly sensitive for diagnosing demyelinating neuropathy [9]. The amplitude, duration, and probability of the occurrence of F-waves (persistence) have considerable roles in gauging the excitability state of motor pools in a variety of central nervous system (CNS) disorders [9,18-20]. In the early stages of ALS, alterations in the excitability of motor neurons correlate with the increased amplitude of the F-wave [20,21]. F-persistence is the method helpful to detect as well as quantify and monitor the progress of the conduction block irrespective of the proximal or distal location [22]. Despite the potential roles of various F-wave parameters, the main application of F-waves is currently limited to latency measurement, particularly F-wave minimum latency which provides information about the conduction properties of the fastest fibers of the F-wave population. Most laboratories use persistence and minimum latency as the only practical measures [13,14]. But as each skeletal muscle receives innervation by two or more nerve roots, the lesion of one root will still keep the minimum latency of the F-wave within normal limits as the other root remains intact. Based on this, it is plausible that the F-wave minimum latency alone may not be a reliable criterion for the early diagnosis of peripheral neuropathy. Several studies have provided evidence of other abnormal F-wave parameters and highlighted their usefulness in cases of peripheral neuropathy [23-25]. The clinical evaluation hence must be based on the laboratory-generated data of F-wave latencies as well as other F-wave parameters. We wish to report the F-wave data in a group of healthy young adults, including uncommonly studied aspects, such as F/M amplitude ratio, duration, chronodispersion, and persistence. The F-wave data in the age group studied can be particularly helpful for the early diagnosis of peripheral neuropathy and the detection of subclinical neuropathies prevalent in the investigated age range. GBS (acute inflammatory demyelinating polyradiculoneuropathy (AIDP)) in which the F-wave has important diagnostic and prognostic roles has young adults often afflicted with. Moreover, studies have reported increased incidences of diabetes mellitus in the youth [26]. The prevalence of DPN was found to be quite high in youth with type 2 diabetes mellitus (mean age: 21.6±4.1 years) [27]. The current data would hence serve to enhance the clinical applicability of several valuable F-wave parameters in various peripheral nervous system disorders in the studied strata.

## Materials and methods

This cross-sectional study was conducted on 100 healthy volunteers in the age group of 18-40 years with normal neurological examinations. The duration of the study was six months (October 2018 to April 2019). The sample size was estimated using Cochran's formula for continuous data, and the participants were selected using the simple random sampling technique [28]. The study protocol was approved by the Institutional Ethics Committee (IEC) of All India Institute of Medical Sciences, Patna (approval number: AIIMS/Pat/IEC/2018/301). The participants studied were the healthy attendants of the patients visiting the Neurophysiology laboratory, and clinical and nonclinical staff of All India Institute of Medical Sciences (AIIMS), Patna, between the ages of 18 and 40 years. The exclusion criteria for the study group were those with peripheral neuropathies, radiculopathies, spastic syndromes, diabetes mellitus, hypertension, and a history of trauma to the limbs.

F-waves were recorded on the Neuro-MEPω EMG and EP digital neurophysiological system software in the Neurophysiology laboratory of AIIMS, Patna. A written informed consent was obtained prior to the study. The participants were given a detailed explanation of the duration, kind, and goal of the study. Information regarding the procedures to be followed was also given to the participants prior to the test. The temperature in the laboratory was maintained at 26°C. F-wave recordings were performed from the abductor pollicis brevis (APB) muscle, abductor digiti minimi (ADM) muscle, abductor hallucis (AH) muscle, and extensor digitorum brevis (EDB) muscle for median, ulnar, tibial, and peroneal nerves, respectively, by surface electrodes (Table 1). The recordings were performed with muscles fully relaxed. The recording and the measurement were performed by a single operator for the entire study. Most subjects were studied bilaterally. However, for the calculation of regression models, data from only one side (non-dominant) were used.

**Table 1:** F-wave recording: electrode placement cm: centimeters

F-wave study (nerve examined)	Recording electrode	Reference electrode	Stimulating electrode
Median	Midpoint of the line between the first metacarpophalangeal joint and the median point of the distal wrist crease, on the most prominent belly of the abductor pollicis brevis muscle	Interphalangeal joint of the thumb	Cathode of the stimulator was placed 8 cm proximal to the recording electrode (measured obliquely along the course of the nerve)
Ulnar	Midpoint of the line between the fifth metacarpophalangeal joint and the pisiform bone	On the middle phalanx of the fifth digit	8 cm proximal to the recording electrode
Tibial	1 cm below the posterior part of the navicular tubercle on the abductor hallucis muscle bulk	First metatarsophalangeal joint or first digit	Immediately behind the medial malleolus
Peroneal	Most prominent part of the extensor digitorum brevis	Fifth metatarsophalangeal joint or fifth digit	At the ankle, just lateral to the tibialis anterior tendon, 8 cm proximal to the recording electrode

**Figure 1:**
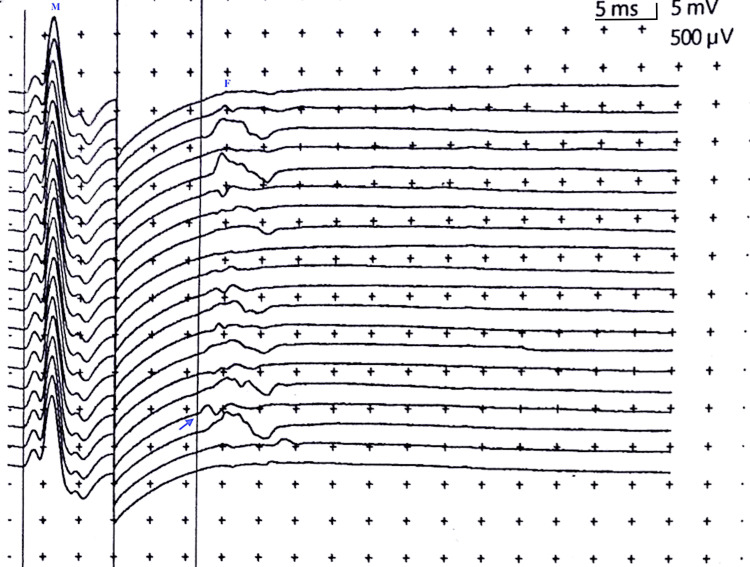
F-wave response recorded from the ulnar nerve (right) in a healthy participant The figure depicts 20 M- and F-responses recorded with supramaximal stimulation and F-wave minimum latency (26.4 ms) (measured as the minimum interval between the stimulus artifact and the first deflection of the late evoked response) (blue arrow). Stimulus frequency: 0.5 Hz; stimulus duration: 0.2 ms; gain: 500 μV/division for F-waves (5 mv/division for M-waves); sweep speed: 5 ms/division Hz: Hertz; ms: milliseconds; μV: microvolts

The stimulus was given at the rate of 0.5 Hz with a stimulus duration of 0.2 ms. The amplifier gain was 500 μV per division, and the sweep speed was 5 ms per division. Stimulation was provided by placing the cathode proximally (positioned at the exact same site utilized for distal stimulation at the wrist or ankle and then anode rotated 180 degrees) [29]. The recommended approach is to utilize a supramaximal stimulus. Few studies, however, have investigated F-wave response using submaximal stimulation which reportedly influenced F-wave persistence and F/M ratio [30]. Supramaximal stimulation was the level of stimulus intensity at which the M-wave amplitude did not further increase. The stimulus intensity employed for eliciting responses was 25% above maximal. Twenty stimuli were provided at supramaximal stimulation. An amplitude of 40 μV or more was accepted [29] (Figure 1).

All F-wave parameters were determined by automated computer algorithms. The latency of the F-response was taken as the interval between the stimulus artifact and the first deflection of the late evoked response. The minimum (FLmn), maximum (FLmx), and mean values (FLmean) were determined for each nerve. Chronodispersion (Fchd) was measured as the difference between the maximum and minimum latency values. F-response duration (FD) was measured between the onset of the deflection from the baseline and the final return to the baseline. F-wave amplitude (FA) was the largest consecutive peak-to-peak amplitude (absolute value). F/M amplitude ratio was obtained as the ratio of mean F-wave amplitude to the maximum corresponding M-response (F/M×100). F-wave persistence (F-prs) was the percentage of F-wave traces with an amplitude of ≥0.5% of the corresponding M-response amplitude/those with ≥40 μV amplitude. As data from such 8-10 identifiable sequential F-waves have been suggested to provide a reasonable estimate of persistence, records with less than 10 traces (with identifiable peak-to-peak amplitude) were not included [31].

Statistical analysis

F-wave minimum latency, F-wave mean latency, F-wave mean duration, persistence, chronodispersion, and F/M amplitude ratio obtained were expressed as mean±standard deviation (SD). Means among the genders were compared by unpaired t-test. Side-to-side comparison was done by paired t-test. For the calculation of regression models, data from only one side were used. The correlation of F-wave parameters with age and height was measured with Pearson's correlation coefficient. A p-value of <0.05 was considered statistically significant. The analysis was conducted using IBM SPSS Statistics for Windows, Version 28.0 (Released 2021; IBM Corp., Armonk, New York, United States).

## Results

One hundred subjects (59 males and 41 females) participated in the study. The mean age of the participants was 28.07±6.37 years (mean±SD). The mean height of the subjects was 164.75±3.36 cm (mean±SD) (Table 2). The ages of the male and female participants were comparable (p=0.61), while height varied in the groups (p=0.0010) (unpaired t-test).

**Table 2:** Anthropometric characteristics of the study population cm: centimeters; n: number of participants; SD: standard deviation

	Age (years) (mean±SD)	Height (cm) (mean±SD)
Male (n=59)	28.35±6.14	165.661±3.02
Female (n=41)	27.68±6.75	163.451±3.44
Total (n=100)	28.07±6.37	164.75±3.36

Bilateral F-wave records for a total of 792 nerves (200 median, 200 ulnar, 198 tibial, and 194 peroneal) in 100 subjects were obtained. Six peroneal nerve records and two tibial nerve records with less than 10 traces with identifiable peak-to-peak amplitude (amplitude ≥40 μV) were not included in the analysis. The values from the two sides were not pooled, since side-to-side comparisons were to be made in the study group.

The values for the F-wave components including mean duration, mean latency, minimum latency, F-wave/M-wave amplitude (F/M ratio), chronodispersion, and persistence for median, ulnar, tibial, and peroneal nerves from both sides as well as side differences (mean±SD) have been depicted in Table 3. 

**Table 3:** F-response: side-to-side differences ***p<0.001; p>0.05 for all other comparisons (paired t-test) Age of the subjects in years (mean±SD): 28.07±6.37; height (cm) (mean±SD): 164.75±3.36 FD: F-wave duration; FLmean: F-wave mean latency; FLmn: F-wave minimum latency; F/M (%): F-wave/M-wave amplitude ratio; Fchd: F-wave chronodispersion; F-prs: F-wave persistence; R: right; L: left; cm: centimeters; n: number of participants; SD: standard deviation; Side diff: side difference

F-wave variable (mean±SD)	Median		P	t	Ulnar		P	t	Tibial		P	t	Peroneal		P	t
	R (n=100)	L (n=100)			R (n=100)	L (n=100)			R (n=99)	L (n=99)			R (n=97)	L (n=97)		
FD (ms)	6.6±0.25	6.64±0.27	0.18	1.35	6.24±0.20	6.25±0.21	0.69	0.39	5.8±0.28	5.82±0.23	0.5	0.68	5.31±0.32	5.34±0.37	0.45	0.76
	Side diff				Side diff				Side diff				Side diff			
	0.04±0.27				0.01±0.28				0.02±0.31				0.03±0.3			
FL mean (ms)	25.49±0.44	25.51±0.47	0.33	0.97	26.46±0.64	26.33±0.72	0.15	1.43	45.18±0.90	45.21±1.05	0.78	0.28	42.89±0.98	42.96±1.0	0.69	0.4
	Side diff				Side diff				Side diff				Side diff			
	0.02±0.23				0.13±0.94				0.03±1.02				0.06±1.37			
FLmn (ms)	24.53±0.69	24.26±0.61	0.0002**	3.84	25.34±1.14	25.03±1.11	0.06	1.92	43.38±1.47	43.28±1.41	0.64	0.46	41.99±1.09	42.16±0.96	0.19	1.33
	Side diff				Side diff				Side diff				Side diff			
	0.27±0.7				0.3±1.6				0.1±2.19				0.18±1.25			
F/M (%)	2.44±0.28	2.45±0.28	0.82	0.22	1.55±0.32	1.53±0.31	0.69	0.39	2.4±0.26	2.39±0.27	0.66	0.44	2.19±0.19	2.21±0.17	0.4	0.84
	Side diff				Side diff				Side diff				Side diff			
	0.009±0.4				0.02±0.44				0.01±0.31				0.02±0.26			
Fchd (ms)	1.88±0.56	1.85±0.53	0.69	0.39	2.52±0.27	2.49±0.26	0.44	0.77	3.6±0.38	3.66±0.30	0.16	1.43	5.39±0.32	5.41±0.33	0.7	0.38
	Side diff				Side diff				Side diff				Side diff			
	0.03±0.74				0.03±0.38				0.06±0.42				0.02±0.49			
F-prs (%)	88.1±6.15	87.3±7.37	0.29	1.05	90.9±6.37	90.4±6.18	0.47	0.73	95.90±2.77	96.40±2.60	0.14	1.47	83.53±3.73	82.81±3.59	0.16	1.4
	Side diff				Side diff				Side diff				Side diff			
	0.8±7.6				0.5±6.87				0.53±3.6				0.7±4.99			

The F-wave minimum latency (mean value) increased by about 0.27±0.7 ms on the right side (as compared to the left) with statistical significance (p<0.001) (paired t-test) for the F-wave median study. No other F-wave components showed significant side-to-side variations for the F-wave response measured (p>0.05) (paired t-test) (Table 3).

**Figure 2:**
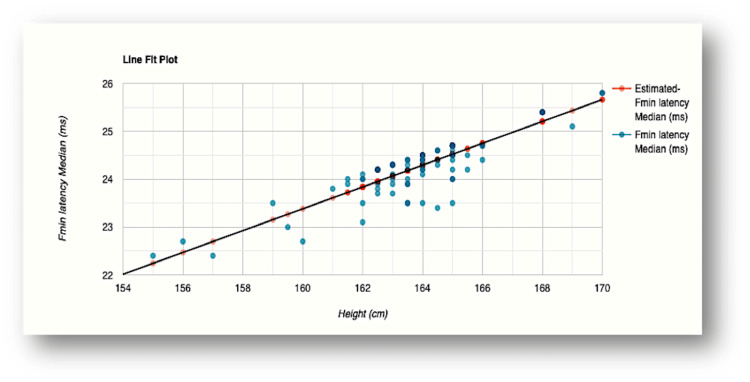
Scatterplot of the relationship between F-wave minimum latency and height for the median nerve R-squared value (R2)=0.7553 (75.5% of the variability of F-wave minimum latency Median (ms) is explained by height (cm) Fmin latency: F-wave minimum latency; ms: milliseconds; cm: centimeters

**Figure 3:**
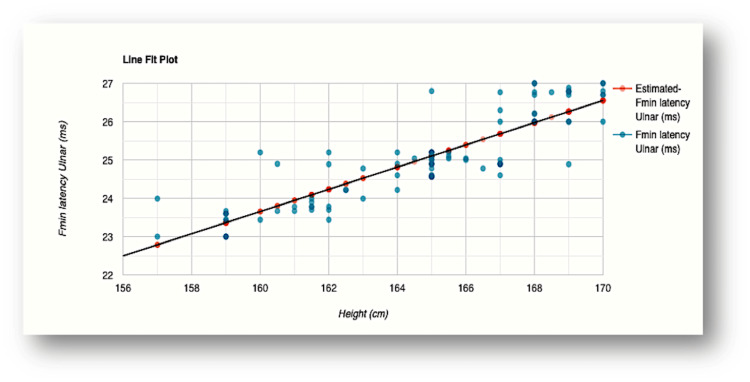
Scatterplot of the relationship between F-wave minimum latency and height for the ulnar nerve R-squared value (R2)=0.7615 (76.1% of the variability of F-wave minimum latency Ulnar (ms) is explained by height (cm) Fmin latency: F-wave minimum latency; ms: milliseconds; cm: centimeters

**Figure 4:**
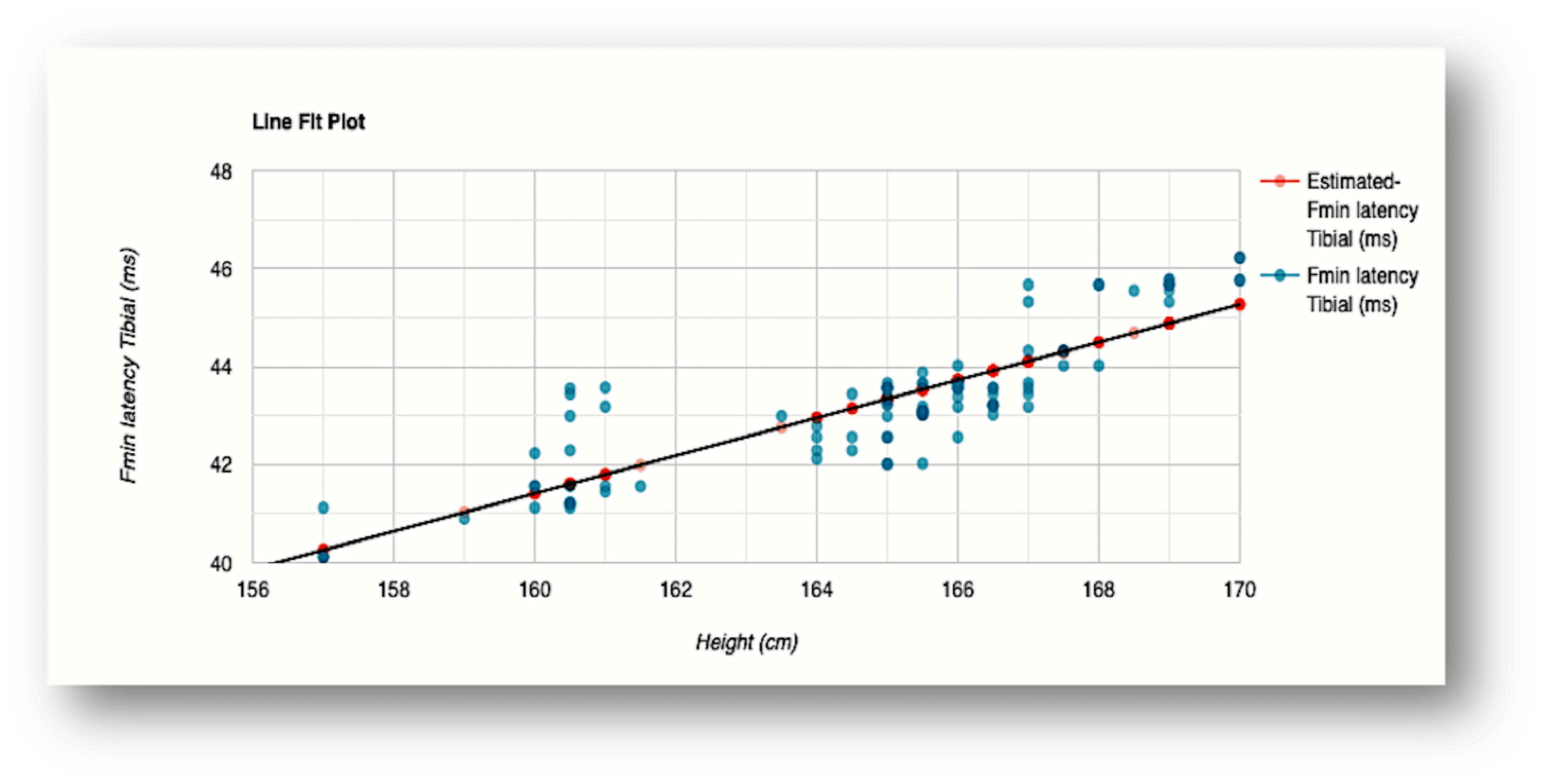
Scatterplot of the relationship between F-wave minimum latency and height for the tibial nerve R-squared value (R2)=0.7439 (74.4% of the variability of F-wave minimum latency Tibial (ms) is explained by height (cm) Fmin latency: F-wave minimum latency; ms: milliseconds; cm: centimeters

**Figure 5:**
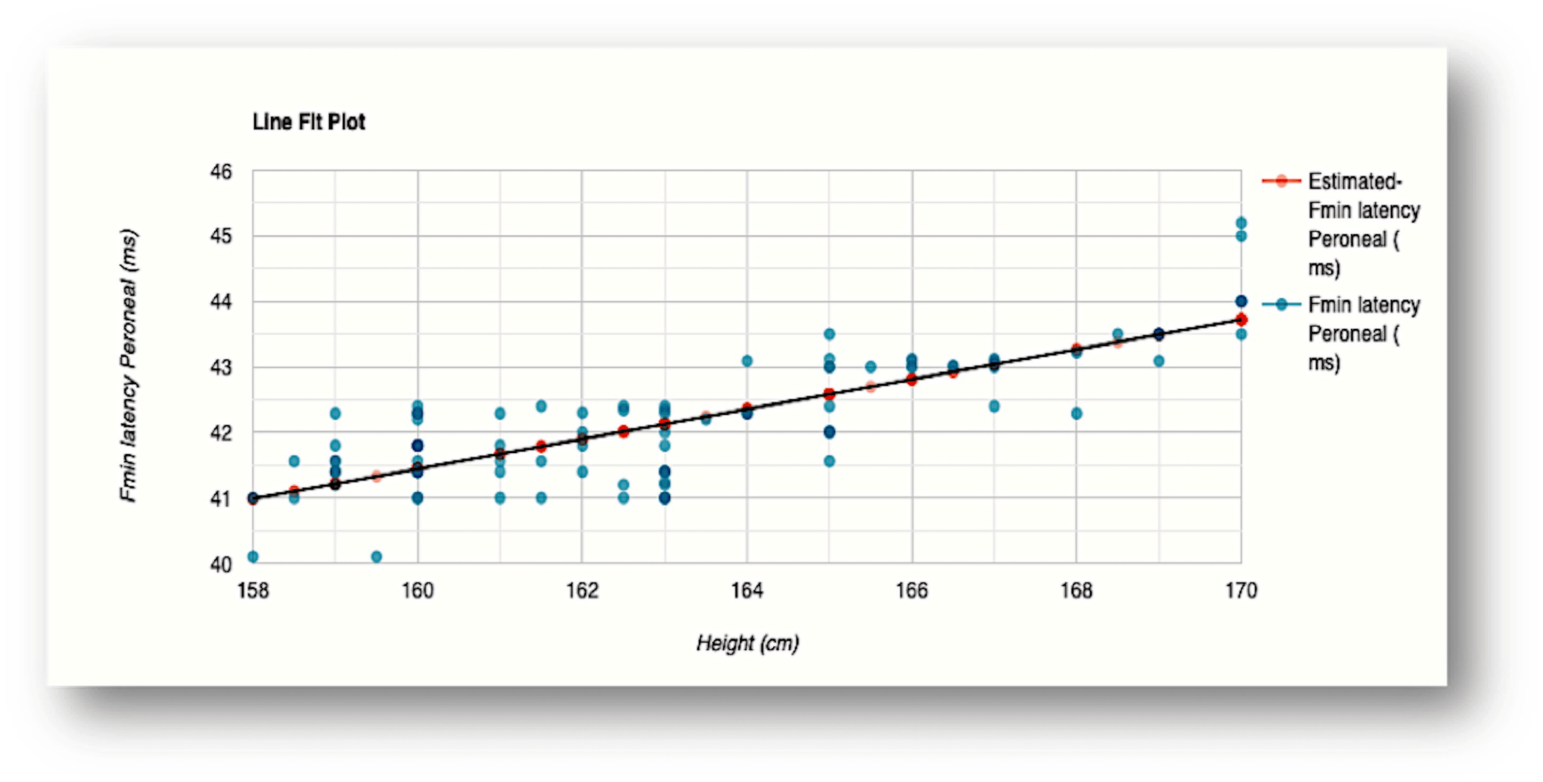
Scatterplot of the relationship between F-wave minimum latency and height for the peroneal nerve R-squared value (R2)=0.6502 (65% of the variability of F-wave minimum latency Peroneal (ms) is explained by height (cm) Fmin latency: F-wave minimum latency; ms: milliseconds; cm: centimeters

The influence of height was evident in F-wave minimum latency values recorded for all the nerves examined (Figures 2-5). The R-squared value (R2) for the variation in F-wave minimum latency (median nerve) by height was 0.7553. This indicated that 75.5% of the variability of F-wave minimum latency (ms) was explained by height (cm) (Figure 2). The R2 for the variation in F-wave minimum latency (ulnar nerve) by height was 0.7615 which indicated that 76.1% of the variability of F-wave minimum latency Ulnar (ms) was explained by height (cm) (Figure 3). In the lower limb nerves, the R2 measured was 0.7439 (F-wave Tibial) and 0.6502 (F-wave Peroneal), indicating 74% and 65% of the variability, respectively, owing to height variations (Figure 4 and Figure 5). The linear regression analysis hence revealed a very strong direct relationship between height (cm) and F-wave minimum latency in all the nerves. When age was entered into the model, the change in the coefficient of determination (R2) was marginal which did not support the effect of age on the variabilities observed. 

The gender difference was evident in the form of increased F-wave minimum latency: p<0.0001 and p<0.01 (unpaired t-test) for median (right and left sides, respectively) while p<0.05 for ulnar, tibial, and peroneal nerves in male subjects (both sides) (Table 4). The F-wave mean latency also increased in male participants but no statistical significance could be attained (p>0.05). No other F-wave component varied among the genders with statistical significance (Table 4). 

**Table 4:** F-response: gender differences ***p<0.0001; **p<0.01; *p<0.05 (unpaired t-test) Males (n=59): age in years (mean±SD): 28.35±6.14; height (cm) (mean±SD): 165.661±3.02 Females (n=41): age (years) (mean±SD): 27.68±6.75; height (cm) (mean±SD): 163.451±3.44 FD: F-wave duration; FLmean: F-wave mean latency; FLmn: F-wave minimum latency; F/M (%): F-wave/M-wave amplitude ratio; Fchd: F-wave chronodispersion; F-prs: F-wave persistence; R: right; L: left; cm: centimeters; ms: milliseconds; n: number of participants; SD: standard deviation

F-wave variable (mean±SD)		Median		Ulnar		Tibial		Peroneal	
		R	L	R	L	R	L	R	L
FD (ms)	Male	6.62±0.27	6.62±0.28	6.26±0.19	6.24±0.2	5.76±0.27	5.80±0.22	5.27±0.32	5.37±0.39
	Female	6.58±0.21	6.67±0.26	6.22±0.21	6.27±0.23	5.85±0.29	5.85±0.23	5.36±0.32	5.28±0.33
P-value		0.46	0.40	0.44	0.52	0.11	0.24	0.17	0.28
t-value		0.74	0.84	0.78	0.65	1.60	1.17	1.39	1.08
FLmean (ms)	Male	25.53±0.42	25.56±0.44	26.47±0.63	26.37±0.72	45.20±0.89	45.28±0.93	42.94±1.01	43.02±1.0
	Female	25.43±0.46	25.45±0.51	26.45±0.66	26.27±0.73	45.14±0.93	45.09±1.20	42.84±0.93	42.86±1.03
P-value		0.23	0.24	0.89	0.47	0.72	0.4	0.64	0.45
t-value		1.20	1.19	0.13	0.72	0.35	0.83	0.46	0.75
FLmn (ms)	Male	24.92±0.47	24.41±0.56	25.55±1.038	25.24±1.09	43.67±1.44	43.51±1.40	42.19±1.12	42.35±0.98
	Female	23.97±0.56	24.05±0.61	25.04±1.22	24.73±1.1	42.95±1.43	42.93±1.38	41.68±0.98	41.87±0.86
P-value		0.0000***	0.0033**	0.027*	0.026*	0.015*	0.04*	0.02*	0.01*
t-value		9.23	3.01	2.24	2.26	2.47	2.04	2.34	2.48
F/M (%)	Male	2.45±0.28	2.45±0.27	1.56±0.33	1.52±0.29	2.38±0.23	2.38±0.26	2.17±0.18	2.22±0.18
	Female	2.42±0.27	2.44±0.28	1.54±0.32	1.55±0.36	2.44±0.29	2.40±0.27	2.23±0.20	2.21±0.15
P-value		0.55	0.86	0.69	0.67	0.25	0.64	0.16	0.71
t-value		0.59	0.18	0.39	0.43	1.16	0.47	1.42	0.37
Fchd (ms)	Male	1.92±0.55	1.84±0.48	2.54±0.26	2.48±0.25	3.61±0.36	3.68±0.29	5.41±0.33	5.42±0.32
	Female	1.83±0.58	1.86±0.61	2.49±0.27	2.51±0.27	3.59±0.39	3.64±0.30	5.35±0.30	5.39±0.35
P-value		0.47	0.87	0.47	0.53	0.74	0.56	0.38	0.7
t-value		0.72	0.17	0.73	0.63	0.33	0.59	0.88	0.38
F-prs (%)	Male	87.80±6.18	87.29±6.91	90.51±6.28	89.66±5.86	95.56±2.69	96.15±2.68	83.49±3.76	82.83±3.71
	Female	88.54±6.15	87.32±8.07	91.46±6.54	91.46±6.54	96.40±2.85	96.85±2.44	83.58±3.72	82.79±3.44
P-value		0.56	0.98	0.46	0.15	0.14	0.19	0.91	0.95
t-value		0.59	0.02	0.73	1.44	1.49	1.32	0.11	0.05

## Discussion

The goal of the current research was to establish quantitative data for relatively rarely studied F-response parameters, as well as to analyze the behavior of various components of the F-response in relation to gender, height, and age. Furthermore, side-to-side comparisons are frequently made in clinical practice for diagnostic evaluation; hence, this was also examined for all F-response components. 

The respective F-response parameters for the right and left sides as well as side-to-side difference values (mean±SD) for all the F-response variables (for median, ulnar, tibial, and peroneal nerves) have been reported in the study (Table 3). The side-to-side differences were not found to be statistically significant for the F-response components in all the nerves examined except for the slight prolongation of the right F-wave minimum latency (0.27 ms±0.7) (p<0.001) (paired t-test) (Table 3) for the F-wave median study. Our findings align well with a previous similar study which stated an increase of 0.3 ms for median F-wave minimum latency on the right side [31]. Studies by Buschbacher and Puksa et al. also reported an increase of 0.2 ms in the right F-wave minimum latency [32,33]. The increased latencies on the right (dominant) side have been explained on the basis of the increased susceptibility to minor trauma in this nerve in the dominant limb, for example, at the wrist within the carpal tunnel [31]. However, such intra-subject variabilities have not been frequent findings in other researches [34,35]. Hence, further evidence to substantiate the current findings would be desirable.

Influence of height, gender, and age on F-wave components

In our study, height explained 75.5% and 76.1% variabilities of minimal F-wave latencies for median and ulnar nerves, respectively, while 74.4% and 65% variations were noted for tibial and peroneal nerves, respectively (Figures 2-5). The regression model in the current study hence depicted that when height (cm) increased by 1, the value of F-wave minimum latency (ms) increased by 0.23, 0.29, 0.38, and 0.23 for median, ulnar, tibial, and peroneal nerves, respectively. Similar findings were obtained for the regression model studied for the influence of height on F-wave mean latency for all the nerves (70.1%, 71.3%, 69.2%, and 64% variabilities in F-wave mean latency explained by height). The current study, hence, supports a strong direct relationship between F-response latency and height for all the nerves examined, as previously reported [31,34,36,37]. The longer F-wave latencies in taller subjects have been explained by two mechanisms. The time taken to travel longer distances in taller subjects has been considered as the first attributing factor. Secondly, slower conduction velocities in taller persons as compared to shorter individuals might contribute to the latency variation [4,38,39].

The influence of gender was most prominently evident as an increase in F-wave minimal latency (with statistical significance): p<0.0001 and p<0.01 (unpaired t-test) for median (right and left sides, respectively) while p<0.05 for ulnar, tibial, and peroneal nerves in male subjects (both sides) (Table 4). An increase in the F-wave mean latency in male participants, however, could not reach statistical significance (p>0.05). Gender differences could not be observed in other F-wave components (Table 4). The findings are in good agreement with those in the previous researches [40,41]. However, longer F-wave latencies in male participants have been explained mainly by height. A similar explanation holds true for the increased F-wave latencies recorded in male participants in the current study. 

F-wave components were not found to vary with age in the current study. Age has been found to have an inconsistent effect when the influence on F-wave components has been studied in the previous similar researches. Height has been reported to explain much more of the variations in F-wave parameters than age [31,33]. Conversely, a previous study in neonates and children has reported latency and amplitude variations with age and has highlighted the role of maturation of myelination for the results obtained [42].

F-wave components

F-wave minimum latency (FLmin) values for upper limb F-response are in accordance with the previous similar researches [43,44]. Puksa et al., however, have recorded slightly lower values (22.9±1.728, 23.7±1.95) for upper limb F-response (median and ulnar nerves, respectively) [33]. On the other hand, Peioglou-Harmoussi et al. have reported F-wave minimum latency to be slightly higher than our values: 27±1.9 and 26.6±1.7 for the right and left median nerve while 27.3±1.8 and 27.1±2.0 for the right and left ulnar nerve, respectively [31]. Lower limb F-responses in our study reveal slightly lower values for tibial and peroneal F-wave minimum latency as compared to previous similar researches [31,43,44]. 

F-wave mean latency (FLmean) values reveal a similar trend as FLmin values when compared with those in past studies [31,43,44]. As there has been a considerable direct relationship between F-response latency and height, differences in the mean height of the participants in various studies might contribute to the mild incongruity observed.

F-wave mean duration (FD) obtained in the current study were 6.6±0.25 and 6.64±0.27 (right and left median nerve), 6.24±0.20 and 6.25±0.21 (right and left ulnar nerve), 5.8±0.28 and 5.82±0.23 (right and left tibial nerve), and 5.31±0.32 and 5.34±0.37 (right and left peroneal nerve) (Table 3). The values are slightly lower when compared with findings from previous studies [31]. Peioglou-Harmoussi et al. have reported mean values of 9.4 ms and 10.1 ms for median and ulnar nerves, respectively [31]. Gencer et al. have reported the F-wave mean duration as 12.45±1.35 ms (tibial nerve) in a control population [35]. A variety of factors have been suggested to influence F-response duration. The most significant of these is the size of the individual motor units, with larger units often having longer durations. Additionally, the number of motor units that are active at the same time and their relative latencies are also relevant [45]. Moreover, the criteria of F-duration measurement have also been different in studies and based on the interval between the onset of the fastest F-response and the end of the slowest F-response (F-"complex" duration) in some studies, and consequently, the findings are not comparable [46]. Interestingly, a negative correlation between F-duration and latency has been reported, and it was concluded that there was an orderly antidromic activation of motor neurons [47]. However, we could not confirm such a correlation in our study. The mean duration has been a sparsely reported parameter in F-response studies so far. According to a previous study, it can have a greater diagnostic value than the other F-wave parameters for the diagnosis of unilateral S1 radiculopathy [35].

F-wave/M-wave amplitude (F/M ratio) obtained in our study were 2.44±0.28 and 2.45±0.28 (right and left median nerve), 1.55±0.32 and 1.53±0.31 (right and left ulnar nerve), 2.4±0.26 and 2.39±0.27 (right and left tibial nerve), and 2.19±0.19 and 2.21±0.17 (right and left peroneal nerve) (Table 3). Our findings well align with the study conducted by Ghosh [48]. Peioglou-Harmoussi et al. have reported median values for F/M amplitude as 1.6 and 1.8 (right and left median nerve) and 1.8 and 2.0 (right and left ulnar nerve) [31]. On the other hand, Parmar and Singh in their report stated higher values [43]. Increases in F-amplitude have been found to be influenced by increases in the size of individual motor units or increased synchronization and/or numbers of motor units making up the F-response and variety of technical and subject characteristics. However, expressing F-response amplitude as a percentage of the simultaneously recorded maximal M-response attenuates these effects. The F/M ratio has been suggested to reflect the resting level of central excitability [49]. With higher motor neuron excitability, the increased number of F-waves will summate to generate large F-waves. Since the M-wave amplitude is unchanged, the F/M ratio may increase in states causing motor neuron hyperexcitability.

Our chronodispersion (Fchd) values conform to those in past studies [32,33,43,44]. Fchd in upper limb F-response is in agreement with that in Puksa et al., while lower limb responses with greater Fchd recorded for peroneal nerve (right and left) (5.39±0.32 and 5.41±0.33) align with those found in Buschbacher and Parmar and Singh [32,43]. The role of Fchd is highlighted by the fact that it has been suggested to be more sensitive than the compound muscle action potential or the F-wave latency measurements in detecting mild neuropathies where affected fibers do not influence the abovementioned parameters owing to the main bulk of unaffected nerve fibers, but F-chronodispersion (which represents the scatter or dispersion of the relative latencies) may be deranged [50]. 

We reported the percentage of stimuli giving rise to F-waves for defining F-persistence (F-prs) and recorded 20 artifact-free traces with identifiable amplitude, which has been commonly used by others and used a similar definition [31-33,51]. F-prs values for the upper limb concord with those reported by Puksa et al., Parmar and Singh, and Mohsen et al. [33,43,52]. Puksa et al. and Parmar and Singh have obtained relatively lower values for lower limb F-prs than ours (Table 3) [33,43]. It has been well identified that F-prs varies between subjects and nerves [22,32]. It ranged from 70% to 100% in normal subjects, and the corresponding values for the peroneal nerve were 17-100% and for the tibial nerve 15-100%. F-prs of the nerves in the upper limb varies from 60% to 100% for the median nerve and from 70% to 100% for the ulnar nerve [2,31]. When interpreting a low F-prs in the peroneal nerve, it has been advised to exercise caution as the presence of only one F-wave is still considered normal in healthy individuals. Conversely, the tibial nerve F-prs is relatively high in normal subjects [33]. 

Limitations

The study did not measure the conduction velocities in the nerves; therefore, we did not attempt to correlate the conduction velocity with the F-wave latencies. The correlation of the weight of the participants with F-response parameters could not be measured. Also, the gender variation observed could have been validated by taking into account the height of the participants in light of the significant height variation among male and female participants. 

## Conclusions

The present study has established age- and gender-specified reference models for the various F-response components including F-wave latencies, F-wave mean duration, F-wave/M-wave amplitude (F/M amplitude ratio), chronodispersion, and persistence in the young adult age group. All the values were found to be in concordance with the literature. Reference values for side differences have also been established. Considering the strong relation of height with F-wave latency parameters, it is important to consider the significant impact of height while interpreting F-response.

By deriving reference values for F/M amplitude ratio, F-wave duration, F-wave persistence, and chronodispersion, which are relatively infrequently studied aspects, the clinical applicability of the F-response study can be enhanced in the identification of pathology.
